# Editorial: Structural biology of nucleic acid replication, recombination and repair

**DOI:** 10.3389/fmolb.2023.1155089

**Published:** 2023-03-15

**Authors:** Edward L. Bolt, Christopher D. O. Cooper, Nicholas P. Robinson

**Affiliations:** ^1^ School of Life Sciences, University of Nottingham, Nottingham, United Kingdom; ^2^ School of Biological and Geographical Sciences, School of Applied Sciences, University of Huddersfield, Huddersfield, United Kingdom; ^3^ CHARM Therapeutics, London, United Kingdom; ^4^ Division of Biomedical and Life Sciences, University of Lancaster, Lancaster, United Kingdom

**Keywords:** replication, recombination, DNA repair, RPA, molecular biology

The 3Rs of replication, recombination and repair of DNA and RNA operate synchronously to ensure that the genomes of all organisms are accurately duplicated, while also responding to DNA damage to guard genomic stability. All of these processes are intimately linked, physically and functionally, by multi-tasking protein complexes that respond to signalling cues when cells need to divide and repair damaged chromosomes. Some of the most direct forms of surveillance for replicative or global genome DNA damage are excision repair complexes—these are able to detect and remove damaged bases (BER enzymes) and nucleotides (NER enzymes) to generate a gap that is filled by fresh DNA replication to replace the damaged DNA. The review article in this volume by Schaich and Van Houten (Pittsburgh, United States) provides a comprehensive discussion of how single molecule biophysical methods have revealed mechanisms for base and nucleotide removal by enzymes from bacteria and eukaryotes. Most impressively, these methods are addressing the question of how BER/NER enzymes distinguish damaged DNA in an expanse of undamaged genomic DNA. Particular highlights are discussions about ‘anomalous diffusion’ as a means for BER/NER enzymes identifying damage, and an overview of the original studies visualising how bacterial UvrA, UvrB, and UvrC can function as a complex to seek out and repair ultraviolet light-induced DNA crosslinks.

Replication, recombination and repair are united in exposing and protecting single strands of DNA in replisomes that separate parental DNA duplex strands, and during DNA end resection and replicative DNA repair required for recombination. DNA single strand binding proteins that achieve this are ubiquitous in nature, often utilizing an OB-fold, but they also show diverse characteristics at interacting with other replication, recombination and repair proteins—they control DNA processing according to signalling cues and cell cycle progression. Analysis by Stuart MacNeill (University of St. Andrews, United Kingdom) of the RPA (replication protein A) family of OB-fold ssDNA binding proteins ventures in detail into archaea. This identifies structural features of archaeal RPA proteins that had eluded classification and understanding from comparisons based on only amino acid sequence homologies. A clear structural distinction between archaeal RPA1-like, and RPA-2-like proteins is presented, and reveals that heterodimers may be important in DNA processing events. The theme of dynamic interactions and oligomeric states of OB-fold containing proteins is continued in the analysis by Piero Bianco (University of Nebraska, United States), who provides a comprehensive review of the structure and functions of OB-folds as “guardians of the genome”, not only when binding to ssDNA but also organising events at Holliday junctions and stalled replication forks. The work also highlights some intriguing unknown OB-fold functions that may link replication, recombination and repair to regulation of other cellular processes.

The diverse and controlling interactions of RPA with other proteins when it is bound to ssDNA are prominent in the review of DNA replication and replisome organisation by Thomas Guillium (MRC LMB, Cambridge, United Kingdom). RPA recruits ligases, ubiquitylases, helicases, and PrimPol (amongst others) that modulate functions of genome stability proteins that restart/re-prime or restrain replication. The different contexts requiring DNA replication are comprehensively reviewed, from stable DNA replication and the latest knowledge about the structure of the replisome through replication fork reversal and translesion synthesis. A collaborative project from Berkeley and Houston in the United States, and KAUST, Saudi Arabia, reported in Tsutakawa eat al., utilises multiple methods to understand potential contributions made by uncharacterised genetic mutations to cancerous tumours, mutations referred to as “variants of unknown significance” (VUS). To gain new understanding of VUS, the groups focussed on those occurring in complexes of RPA with helicase and nuclease enzymes that are known to be critical for the biology of cancers. The result is an elegant combination of modelling drawn from data about molecular evolution, atomic resolution structures of the protein complexes, and known outcomes from groups of VUS in human cells.

When recombination is not possible at a DNA break the DNA repair apparatus of non-homologous end-joining (NHEJ) takes over to re-join together the broken DNA ends. The review article led by Amare et al. (MacMaster University, Canada) focusses on bacterial NHEJ and the remarkable multi-tasking abilities of the enzyme LigD. This chemically modified the DNA ends and ligates them, aided by interactions with Ku proteins. In this review the details of each experimentally determined LigD structure are provided and scrutinised, revealing that the catalytic cores of LigD enzymes gain additional functional characteristics from accessory regions that are diverse across bacteria, and also revealing some intriguing features of unknown function. The crucial role of NHEJ in bacterial survival from DNA breaks, allied with the widespread use of DNA-break causing antibiotics that target DNA replication, makes a full understanding of the molecular details of NHEJ in bacteria significant for developing new targets and understanding potential antibiotic resistance mechanisms.

This Volume 1 of articles highlights molecular mechanisms of DNA recombination, replication, repair in bacteria, archaea and eukaryotes, and how they contribute to understanding the molecular basis of processes that impact on human health. We look forward to opening Volume 2 for which we invite contributions about the structure and function of replication, recombination and repair in all clades of life, including viruses.

